# Changing seroprevalence of schistosomiasis japonica in China from 1982 to 2020: A systematic review and spatial analysis

**DOI:** 10.1371/journal.pntd.0012466

**Published:** 2024-09-03

**Authors:** Yu Zhou, Mao Zheng, Yanfeng Gong, Junhui Huang, Jiamin Wang, Ning Xu, Yixin Tong, Yue Chen, Qingwu Jiang, Yu Cai, Yibiao Zhou

**Affiliations:** 1 School of Public Health, Fudan University, Shanghai, China; 2 Key Laboratory of Public Health Safety, Fudan University, Ministry of Education, Shanghai, China; 3 Fudan University Center for Tropical Disease Research, Shanghai, China; 4 Hunan Institute for Schistosomiasis Control, Yueyang, Hunan Province, China; 5 School of Epidemiology and Public Health, Faculty of Medicine, University of Ottawa, Ottawa, Canada; Federal University of Agriculture Abeokuta, NIGERIA

## Abstract

**Background:**

Schistosomiasis is a global public health issue. In China, while the seroprevalence of Schistosomiasis japonica has currently reduced to a relatively low level, risk of infection still exists in certain areas. However, there has been a lack of comprehensive research on the long-term trends of national seroprevalence, changes across age groups, and characteristics in spatial distribution, which is crucial for effectively targeting interventions and achieving the goal of eliminating schistosomiasis by 2030. Our study aimed to address this gap by analyzing the long-term trends of Schistosomiasis japonica seroprevalence in China from 1982 to 2020 based on the data from diverse sources spanning a period of 39 years.

**Methodology:**

Seroprevalence data were collected from literature databases and national schistosomiasis surveillance system. Meta-analysis was conducted to estimate the seroprevalence. Joinpoint model was used to identify changing trend and inflection point. Inverse distance weighted interpolation was used to determine the spatial distribution of seroprevalence.

**Principal findings:**

The seroprevalence decreased from 34.8% in 1982 to 2.4% in 2020 in China. Before 2006, the seroprevalence was higher in the middle age group, and a pattern of increasing with age was observed afterwards. The areas with high seroprevalence existed in Dongting Lake, Poyang Lake, Jianghan Plain, the Anhui branch of the Yangtze River and some localized mountainous regions in Sichuan and Yunnan provinces.

**Conclusions/Significance:**

There was a significant decline in the seroprevalence of Schistosomiasis japonica from 1982 to 2020 in China. Nevertheless, schistosomiasis has not been eradicated; thus, implementing precise and personalized monitoring measures is crucial for the elimination of schistosomiasis, especially in endemic areas and with a particular focus on the elderly.

## Author summary

Serological immunological testing is a crucial means of monitoring schistosomiasis. In China, while the seroprevalence of Schistosomiasis japonica has currently reduced to a relatively low level, risk of infection still exists in certain areas. Achieving the goal of eliminating schistosomiasis by 2030 necessitates more precise and effective prevention and control measures. Our study intended to figure out the long-term trends of Schistosomiasis japonica seroprevalence in China from 1982 to 2020, focusing on age and spatial variations to identify high-risk areas and populations. We expect that the results can inform the development of targeted measures for schistosomiasis elimination.

## 1.Background

Human schistosomiasis is one of the most important neglected tropical diseases (NTDs) [1] that affects approximately 250 million people worldwide, leading to an estimated burden of 3.3 million disability-adjusted life-years (DALYs) annually [2,3]. This chronic parasitic disease is endemic across 78 countries and regions in Africa, Asia, and Latin America [4], with approximately 779 million people at risk of acquiring the infection [5].

There are three main species of schistosomes that can infect human beings [6]. In China, all indigenous cases are caused by infection with the species *Schistosoma japonicum (S*. *japonicum)* [7]. Schistosomiasis may has had a history of over 6000 years in China and was once the most severe helminth infection in the country [8]. Over the past 70 years, successful schistosomiasis control programs have been implemented in China, such as large-scale elimination of snails, synchronous chemotherapy for humans and animals, and controlling all sources of transmission [9]. These measures have dramatically reduced the prevalence of schistosomiasis [10]. Currently, the schistosomiasis epidemic situation remains at a relatively low level, with 75.9% of the endemic counties achieving the criteria of elimination by 2022 [11]. The Chinese government has set an ambitious goal of eliminating schistosomiasis nationwide by 2030 [7].

The effective control and prevention of schistosomiasis necessitates reliable diagnostic methods to precisely identify potential target population for treatment [12]. The WHO recommends the Kato-Katz (KK) technique to confirm intestinal schistosomiasis infection [13]. However, with the evident decline in schistosomiasis prevalence, this fecal examination test is no longer suitable due to its low sensitivity [14]. With the development of immunological and molecular biology technologies, some diagnostic methods based on detecting anti-schistosome antibodies in human serum have demonstrated increased sensitive and time-efficient, particularly suitable for disease monitoring [15,16]. Regions with a higher seroprevalence may indicate a risk of schistosomiasis transmission. China has incorporated the immunodiagnostic technology into the schistosomiasis control program since early 1980s to improve diagnostic records and identify target individuals for treatment [17].

Previous research on the seroprevalence of schistosomiasis in China mainly focused on some specific populations [18,19], changing trends in localized regions [20], and environmental and socio-economic influencing factors [4]. To a certain extent, the control of schistosomiasis relies on effective implementations of various national control strategies [21]. However, there has been a lack of comprehensive research on the long-term trends of national seroprevalence, changes across age groups, and characteristics in spatial distribution, which is crucial for effectively targeting interventions and achieving the goal of eliminating schistosomiasis by 2030. Our study aimed to address this gap by analyzing the changes in the seroprevalence of schistosomiasis across various periods, age groups, and spatial locations since the implementation of immunodiagnostic tests in China based on the data from diverse sources from 1982 to 2020. Due to the focal pattern of distribution of schistosomiasis [21], we collected data at the village level, the smallest administrative units in China. This information may inform the development of targeted measures for schistosomiasis elimination.

## 2. Materials and methods

### 2.1 Data sources and collection

#### 2.1.1 Search strategy

We searched PubMed, Web of Science, China National Knowledge Infrastructure (CNKI, https://www.cnki.net/), Wanfang Database (https://www.wanfangdata.com.cn/) and VIP Chinese Journal Database(VIP, http://qikan.cqvip.com) for articles on the seroprevalence of *S*. *japonicum* infection in China from January 25, 1983 to September 27, 2023. We applied the search terms “schisto* (OR bilhar*) AND China (OR Chinese) AND survey (OR screen, OR surveillance, OR monitor) AND prevalence (OR positivity, OR sero*, OR serum OR immun*)” without any other restrictions (e.g., survey date or study design). We also checked relevant literature manually by searching the reference lists. We initially screened titles and abstracts to identify potential articles and removed duplicates. We excluded case reports, in vitro studies, non-human studies, or schistosomiasis not reported. We then filtered the articles using the following exclusion criteria: (1) The survey time was unclear, lacking specificity to a particular year; (2) The study results were aggregated within regions, not specifically focusing on the village or community level; (3) the number of people screened and the number of seropositive cases were not given, or they couldn’t be obtained through the calculation using the formula (seroprevalence = number of seropositive cases / number of people screened); (4) the surveyed individuals can not represented the general population of the local area (e.g. patients with a given disease, immigrants, travelers, military personnel); (5) case–control studies, clinical trials, drug-efficacy studies and interventions, and review articles; (6) full text is not available. If two articles published the same research data, the article with less information was excluded. Since there were few articles reporting the seroprevalence after 2020, our research focused on the period from 1982 to 2020.

#### 2.12. Data extraction

Two researchers independently conducted a preliminary screening of each identified title and abstract, and then read the full text for a second screening. We collected the following information from the extracted references: number of people screened, seroprevalence (or number of seropositive cases), age group, immunological diagnostic method used, survey time (year), study location (province, villages). We also obtained 66 records from the national schistosomiasis surveillance spanning the period from 2000 to 2004. The coordinates for the survey locations were obtained from Google Earth due to its comprehensive and easily accessible global satellite imagery, which provides a consistent and high-resolution dataset suitable for our research needs. We also corrected the coordinates for offset.

### 2.2. Statistical analyses

#### 2.2.1. Meta-analysis

We conducted a subgroup analysis by survey year to calculate the overall seroprevalence for each year from 1982 to 2020 and the age-specific seroprevalence. The heterogeneity between studies was assessed using Cochran’s Q (chi-square) test and quantified by Higgins inconsistency statistic (*I*^*2*^) [22]. *I*^*2*^ indicates whether a difference between studies is caused by heterogeneity rather than chance, and the values of 25%, 50% and 75% correspond to low, medium, and substantial heterogeneity [23]. When there was substantial heterogeneity (*I*^*2*^ > 50%), a random effect model was used to combine seroprevalence estimates; otherwise, a fixed effect model was used [24]. To assess the robustness of the annual pooled seroprevalence, we conducted a sensitivity analysis by sequentially omitting the studies with the largest and smallest sample sizes for each year, and then reanalyzed the seroprevalence for each year. If the exclusion of the study did not result in the pooled estimate falling outside the 95% confidence interval of the original pooled seroprevalence, it was considered that the sample size had no impact on the results [25,26]. All the meta-analyses were performed with Stata18.0 (Stata Corp., College Station, Texas, USA).

#### 2.2.2. Changing trends of seroprevalence

We used Joinpoint model (Joinpoint Regression Program 5.0.2, National Cancer Institute, Rockville, MD, USA) to explore the changing trends of seroprevalence for the period between 1982 and 2020. We selected logarithmic linear model for trend to calculate the annual percentage change (APC) and its 95% CI [27]. APC reflects the direction and speed of the trend change. APC< 0 indicates that the seroprevalence decreases with time, APC> 0 indicates that the seroprevalence increases with time [28].

#### 2.2.3. Surface interpolation to estimate mean seroprevalence

To explore the spatial characteristics of seroprevalence in different time periods, survey years were divided into three periods: prior to 2000, 2000–2009 and 2010–2020. We averaged the seroprevalence at the same location in the same period and then used inverse distance weighted (IDW) interpolation to estimate the mean seroprevalence. Furthermore, to explore the spatial distribution of seroprevalence among individuals under 20 years old, we calculated the average seroprevalence at each location throughout the study period due to limited sample size in this age group. We then employed the IDW interpolation method to estimate the average seroprevalence under 20 age group.

IDW is a deterministic spatial interpolation method, which takes the spatial distance between object points as a weight [29]. The closer the distance is, the greater the weight is [29]. The interpolation analysis was performed with ArcGIS 10.8 software (Esri, Redlands, CA, USA).

## 3. Results

### 3.1. Data of seroprevalence included in the current analysis

**Fig 1:**
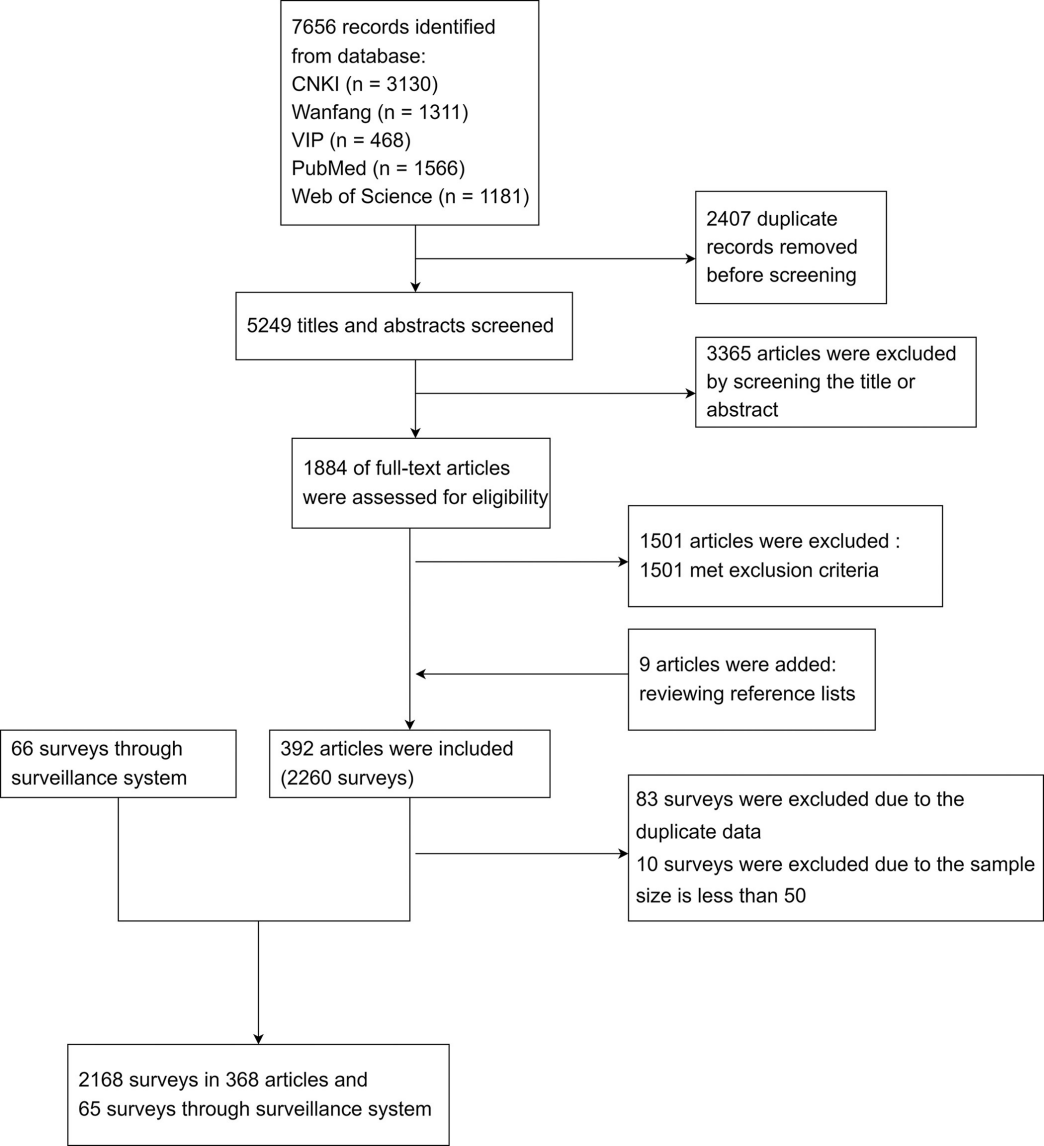
Flow chart of search and selection of articles.

A total of 392 articles met the selection criteria (Fig 1). In addition, 66 surveys were identified from the national surveillance database, 83 duplicate surveys and 10 surveys with the sample size less than 50 were excluded (Fig 1). Finally, the analysis included 2168 surveys from 368 research articles and 65 surveys from the surveillance. The data spanned from 1982 to 2020, covering 752 independent villages or communities in nine schistosomiasis endemic provinces in China (S1 Table).

### 3.2. Changing trend of annual seroprevalence

**Fig 2:**
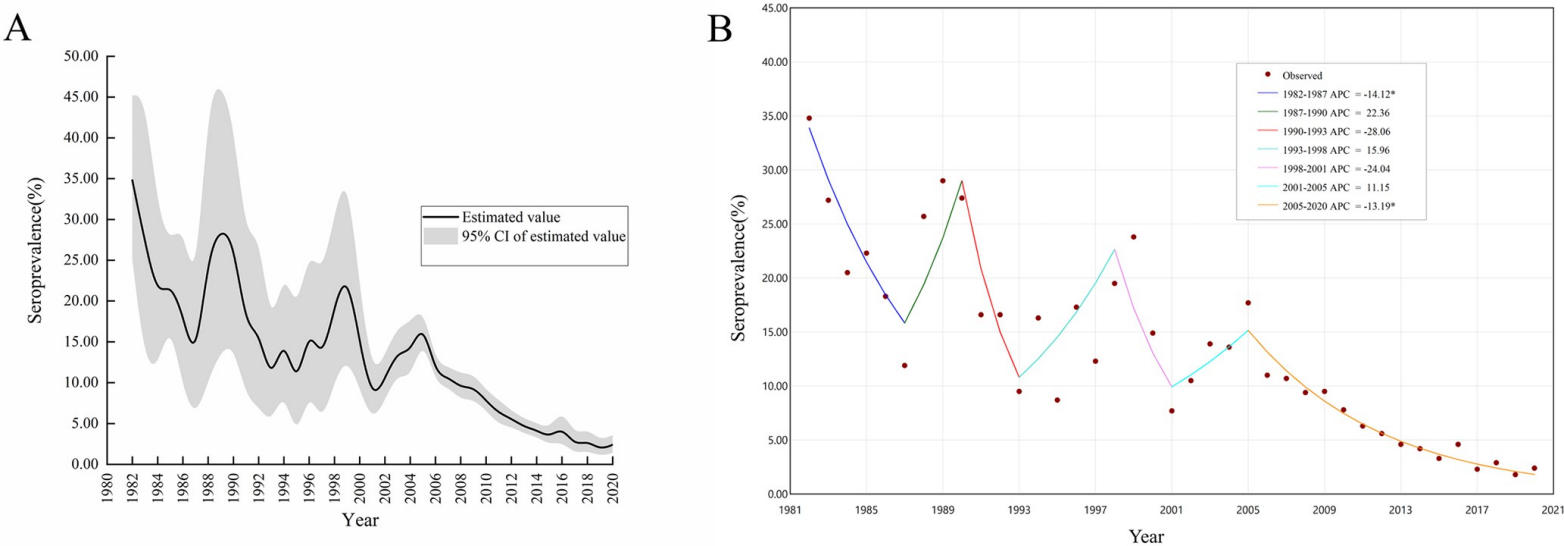
Changing trend of seroprevalence between 1982 and 2020. (A: The estimation and 95%CI of the seroprevalence; B: Joinpoint analysis for the seroprevalence, *denotes *P* < 0.05; *APC*: Annual percentage change).

Since there was heterogeneity between studies (Q = 2.7e+05, *P*<0.001, *I*^2^ = 99.17%) (S3 Fig), likely due to the inclusion of studies from different regions and various time periods, a random effect model was used to estimate the seroprevalence. The sensitivity analysis indicated that after excluding the studies with the largest or smallest sample sizes, the estimated annual seroprevalence remained within the 95% confidence interval (CI) of the original pooled seroprevalence for each year (S1, S2 and S3 Figs). This demonstrated that the pooled seroprevalence was not substantially affected by the sample size. The stability of these results validated the reasonableness and reliability of our analysis. The annual seroprevalence showed a decreasing trend and declined significantly from 34.8% (95% CI: 25.1%, 45.2%) in 1982 to 2.4% (95% CI: 1.4%, 3.6%) in 2020 (Fig 2A and S3 Fig). The seroprevalence showed a notable fluctuation for the period between 1982 and 2005 and then declined steadily from 2005 (17.7%, 95% CI: 15.8%, 19.6%) to 2020 (2.4%, 95% CI: 1.4%, 3.6%).

For the period between 1982 and 2020, the Joinpint model identified six inflection points (1987, 1990, 1993, 1998, 2001, 2005) and two of them are statistically significant (1987, 2005) (Fig 2B). The seroprevalence showed a fluctuant and significant downward trend from 1982 to 2001. After 2005, the seroprevalence has consistently declined with an APC of– 13.19% (95% CI: − 14.47%, −11.90%) (Fig 2B).

### 3.3. Changing trend of seroprevalence in age groups

**Fig 3:**
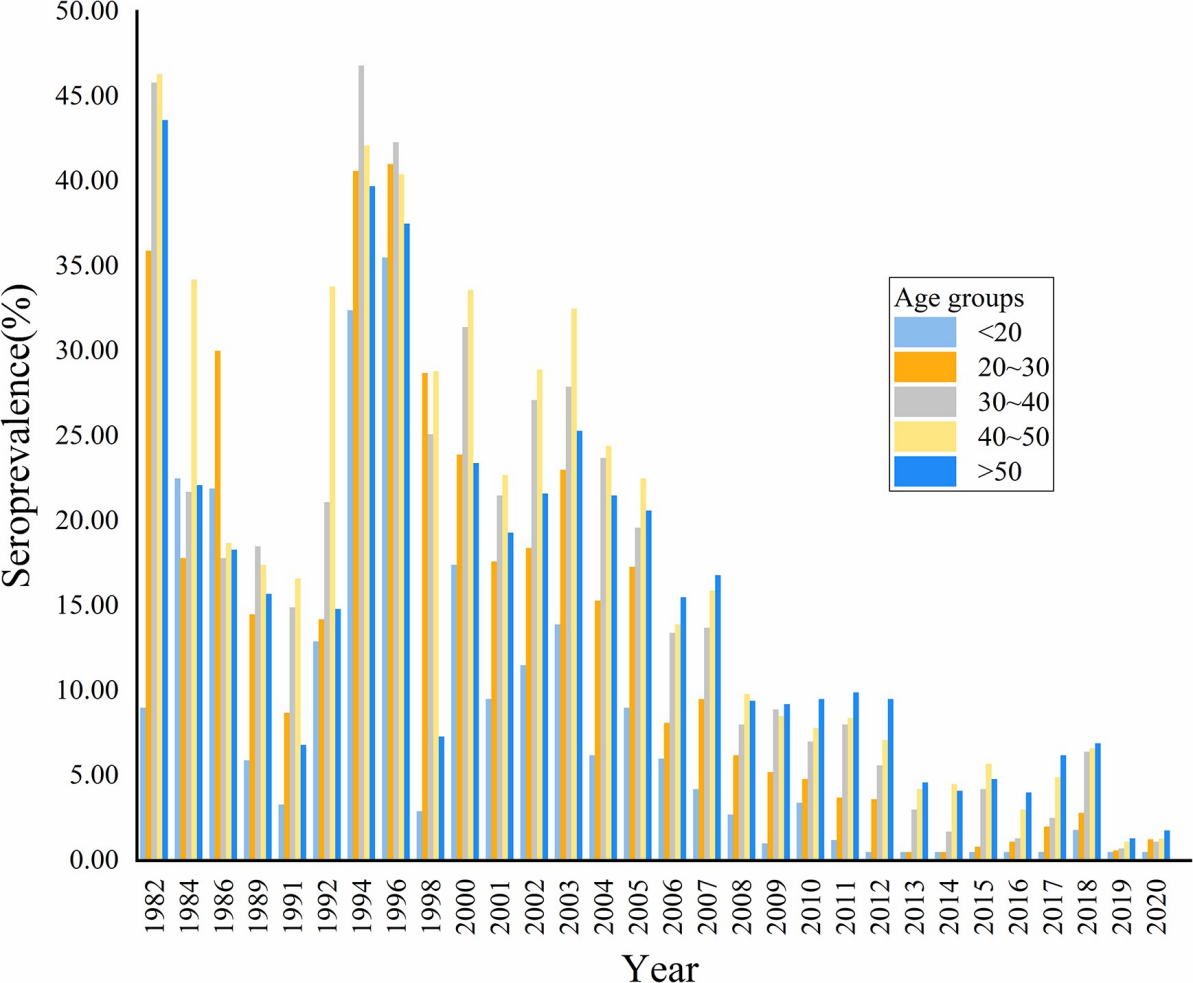
The seroprevalence of schistosomiasis japonica in different age groups.

Fig 3 showed the age-specific seroprevalence according to calendar year. Before 2006, the seroprevalence was higher for the age groups of 30–40 and 40–50 compared with the younger (<20) and older (>50) age groups. Starting from 2006, the seroprevalence increased with age. Overall, the seroprevalence showed a fluctuating downward trend across different age groups and remained below 2.0% in all age groups since 2019 (Fig 3 and S2 Table).

### 3.4. Spatial distribution of seroprevalence

**Fig 4:**
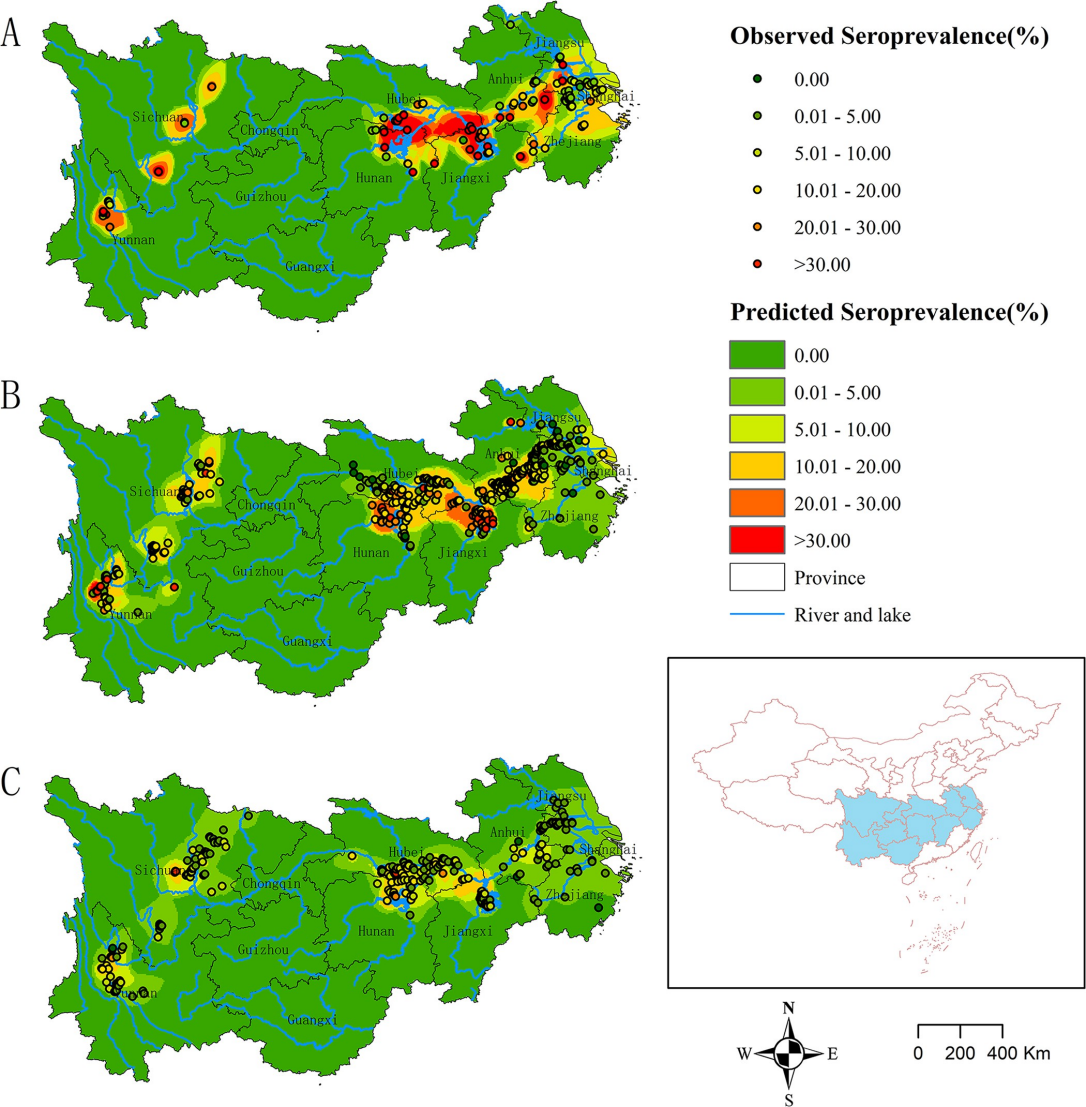
Spatial distribution of seroprevalence in China between 1982–2020 based on inverse distance weighted interpolation. (A: Before 2000; B: During 2000–2009 C: During 2010–2020). The source of the basemap shapefile was from the National Catalogue Service For Geographic Information (www.webmap.cn).

**Fig 5:**
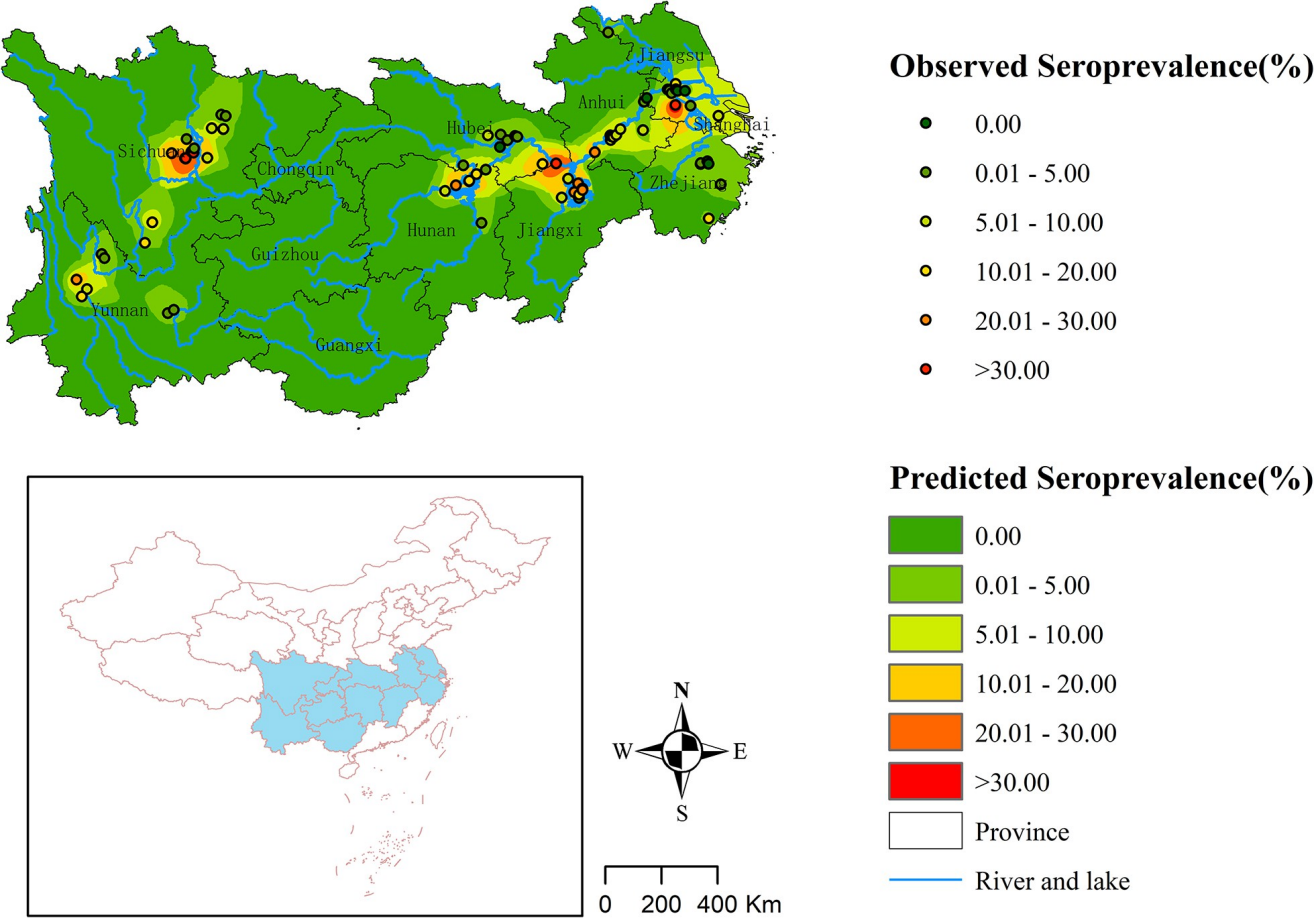
Spatial distribution of the average seroprevalence under 20 years old from 1982 to 2020. The source of the basemap shapefile was from the National Catalogue Service For Geographic Information (www.webmap.cn).

There were 106, 499, and 253 independent villages that conducted serological screenings before 2000, between 2000 and 2009, and between 2010 and 2020 respectively (Fig 4). IDW showed that the seroprevalence decreased nationwide over the past four decades (Fig 4). Before 2000, the endemic areas of schistosomiasis were primarily situated around the two major freshwater lake basins in China (Dongting Lake and Poyang Lake), and along the Yangtze River and its tributaries in Hubei, Anhui, Zhejiang, and Jiangsu provinces, as well as in some mountainous regions of Yunnan and Sichuan provinces. From 2000 to 2009, there was a noticeable decline of seroprevalence in Zhejiang and Jiangsu provinces; however, schistosomiasis was still prevalent in Hunan, Hubei, Anhui, Jiangxi, Yunnan, and Sichuan provinces. After 2010, the national seroprevalence reached a low level. During this period, the endemic areas primarily clustered around Dongting Lake and Poyang Lake basins, Jianghan Plain, the Anhui branch of the Yangtze River, and some localized mountainous regions in Yunnan and Sichuan provinces (Fig 4). The spatial distribution of seroprevalence among the younger age group aligned closely with the general population distribution, but with a more confined range in high-risk regions (Fig 5). Over the past four decades, areas with higher seroprevalence among the younger age group were primarily concentrated in the Dongting Lake basin, the border region between Hubei Province and Jiangxi Province, as well as local areas of Sichuan Province and Jiangsu Province (Fig 5).

## 4. Discussion

This study explored the temporal, age, and spatial characteristics of the seroprevalence of *S*. *japonica* in China over the past four decades. The seroprevalence demonstrated a fluctuating downward trend between 1982 and 2020, mainly due to various schistosomiasis control measures.

The seroprevalence declined from 34.8% in 1982 to 11.9% in 1987. However, it then experienced an upward trend, reaching 29.0% in 1989 and 27.4% in 1990. From 1990 to 2001, the seroprevalence showed a fluctuating downward trend, followed by a subsequent increase, reaching a peak of 17.7% in 2005 and gradually declining thereafter. Before 1985, the strategy of schistosomiasis control in China primarily focused on eliminating snails [30], and as a result, the reduction in snail habitats and the number of infected people was significant [31]. However, this strategy had disadvantages of high costs and lack of effective control over the sources of infection [32], with a low sustainability. In 1989, an outbreak of acute schistosomiasis occurred on Yangyuan Street of Wuhan City due to a prolonged duration of flood season of Yangtze River [33], and our research also showed that the seroprevalence reached a peak in 1989 and 1990. In response to the resurgence of schistosomiasis, the State Council of China proposed supportive measures such as chemotherapy in severely affected areas [33]. Subsequently, in 1992, the World Bank Loan Project (WBLP) for controlling schistosomiasis was implemented in eight provinces in China [34]. These might explain the lower seroprevalence observed in our study in 1993. The WBLP was completed in Anhui, Jiangsu, Jiangxi, Sichuan and Zhejiang at the end of 1998, and continued until the end of 2001 in Hubei, Hunan and Yunnan [35,36]. During the WBLP period, chemotherapy with praziquantel was widely implemented in China. However, after multiple rounds of mass or selective treatment, the compliance rates for chemotherapy decreased in many communities. Coupled with the flooding events of 1998, these factors may have contributed to the higher prevalence in 1998 [10,35]. Although the project stabilized the prevalence of schistosomiasis [37], the morbidity control based on chemotherapy failed to prevent the re-infection of schistosomiasis in humans and livestock. Therefore, a resurgence of schistosomiasis occurred following the termination of WBLP, particularly after severe flooding in the Yangtze River Basin in 1998 [38], causing a significant increase in the prevalence of schistosomiasis between 2002 and 2005. A new integrated strategy focusing on infection source control had been carried out since 2005 [39] and was highly effective to reduce the transmission of schistosomiasis [39–42]. Our research further highlighted a marked decrease in seroprevalence after 2005, evidencing the enduring success of this comprehensive strategy that includes source management of schistosomiasis, patient chemotherapy, snail control, health education, improved sanitation, and safe management of drinking water, etc. Therefore, sustained control efforts, particularly those focusing on infection source control, have the potential to significantly reduce the burden of schistosomiasis, contributing to the goal of schistosomiasis elimination.

The changes in seroprevalence across different age groups primarily manifested in two aspects. On one hand, consistent with the overall trend, seroprevalence across different age groups has shown a fluctuating decline over time, with the age groups under 20 and 20–30 experiencing the most significant decreases. After 2010, both these age groups reached relatively low levels of seroprevalence, with the under 20 age group mostly staying below 1% and the 20–30 age group maintaining around 2%. On the other hand, before 2006, seroprevalence was higher in the middle age groups, but since 2006, it has increased with age.

Firstly, schistosomiasis is water-borne helminthiasis [43]. The observed patterns can be largely attributed to shifts in occupational and demographic dynamics across different age groups in rural China. Due to the shift in occupation from agriculture to urban industrial work among young people, coupled with improved educational levels, which consequently reduced contact with infected waters and led to a significant decrease in seroprevalence [44]. This occupational shift had also changed the age structure of the rural population, resulting in an aging population and labor force in rural areas. Additionally, with relatively fixed occupations and living environments, the decline in seroprevalence was more limited among the older age groups [45,46]. Secondly, antibodies can persist in the human serum for 2–3 years or even longer after chemotherapy with praziquantel [47,48]. The decline of anti-schistosome antibodies is associated with age, and serum antibodies fade faster in the younger age groups than in the older ones [49]. Additionally, the changes of specific antibody levels after praziquantel treatment demonstrated heterogeneity related to host’s age [50]. Given the variations in seroprevalence among different age groups, future control measures need to be more targeted, particularly in implementing protective measures for the elderly in rural areas to reduce infection among these high-risk populations. Additionally, future research should concentrate on developing personalized monitoring methods for different age groups, enhancing the effectiveness of schistosomiasis control.

The spatial distribution of schistosomiasis showed no substantial changes over the 39-year period. The endemic areas of both the total population and the younger age group concentrated in two mountainous provinces and seven provinces along the middle and lower Yangtze River [8]. The overall seroprevalence of schistosomiasis decreased nationwide. Among the 253 surveyed villages from 2010 to 2020, only 3 villages had a seroprevalence exceeding 30.0%, while 141 (55.7%) villages had a seroprevalence below 5.0%. Currently, schistosomiasis endemic areas are predominantly located in the Dongting and Poyang Lake regions, Jianghan Plain, the Anhui branch of the Yangtze River, as well as localized mountainous areas. The two major lake regions have a higher snail density [28], a larger population of boatmen, and a higher prevalence of schistosomiasis in wild animals [51], making it difficult to completely eliminate the sources of infection. Consequently, it has formed a complex and challenging-to-control endemic areas of schistosomiasis. In mountainous and hilly endemic areas, it is also difficult to eliminate schistosomiasis due to geographical factors and population mobility [52]. To achieve the goal of elimination of human schistosomiasis by 2030, the government should implement precise prevention and control measures tailored to the unique ecological and socio-economic conditions of different endemic areas, especially among the elderly people and wild animals in these endemic areas.

This study has some limitations. Firstly, only 263 surveys were collected before 2000, and the limited data may lead to unstable estimates of seroprevalence for certain years. Secondly, serological testing has its limitations, such as its inability to distinguish between recent and former infections for schistosomiasis [4]. In areas where schistosomiasis coexists with other parasites, the specificity of serological testing may be compromised due to cross-reactivity [53]. To enhance the comprehensiveness of our data, the studies we included employed various serological testing methods to assess seroprevalence, each differing in sensitivity and specificity. The methodological diversity may lead to variability in results, potentially impacting our overall conclusions.

Our data may overlap with the annual reports from the China CDC for certain years. While the China CDC annual reports only provide a broad, provincial-level overview, our study offers more granular data and covers a broader time range, including detailed analyses by age group and spatial distribution. Overall, our findings highlight significant trends and regional differences that can inform targeted public health interventions. Future research should incorporate updated data and employ improved methodologies to further validate and expand our findings, thereby enhancing the effectiveness of schistosomiasis control and prevention strategies.

## Supporting information

S1 Table The list of included studies. (XLSX)

S2 Table Age group data. (XLSX)

S1 Fig Sensitivity analyses of seroprevalence with the largest sample size study omitted. (TIF)

S2 Fig Sensitivity analyses of seroprevalence with the smallest sample size study omitted. (TIF)

S3 Fig Forest plot of annual seroprevalence for schistosomiasis with random-effects analyses. (TIF)
