# Supplementary figures and images for: Changing seroprevalence of schistosomiasis japonica in China from 1982 to 2020: A systematic review and spatial analysis
Source: PLoS Negl Trop Dis. 2024 Sep 3;18(9):e0012466. doi: 10.1371/journal.pntd.0012466 (PMC11398675; doi:10.1371/journal.pntd.0012466)

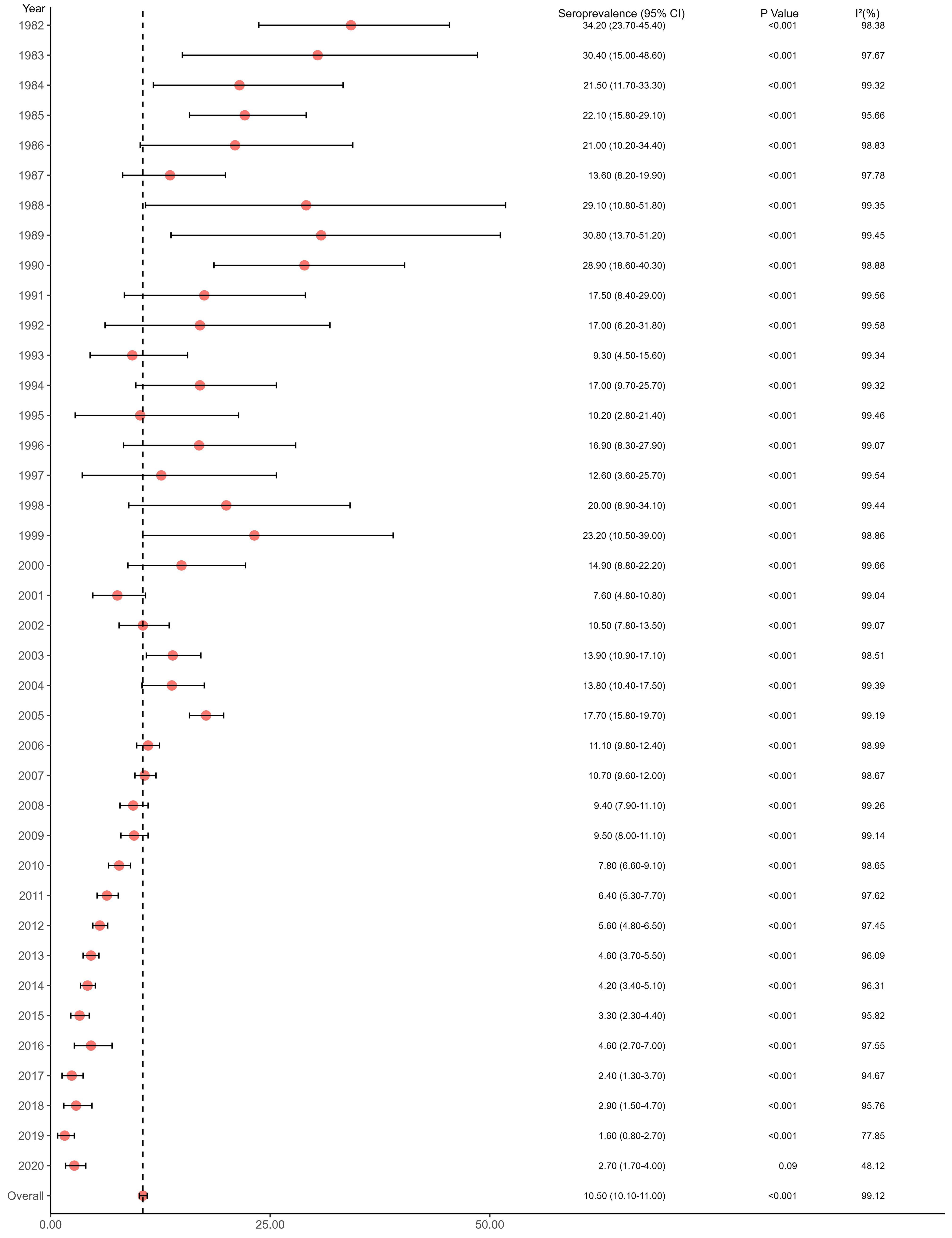

Supplement: S1 Fig — (TIF) [file pntd.0012466.s003.tif]

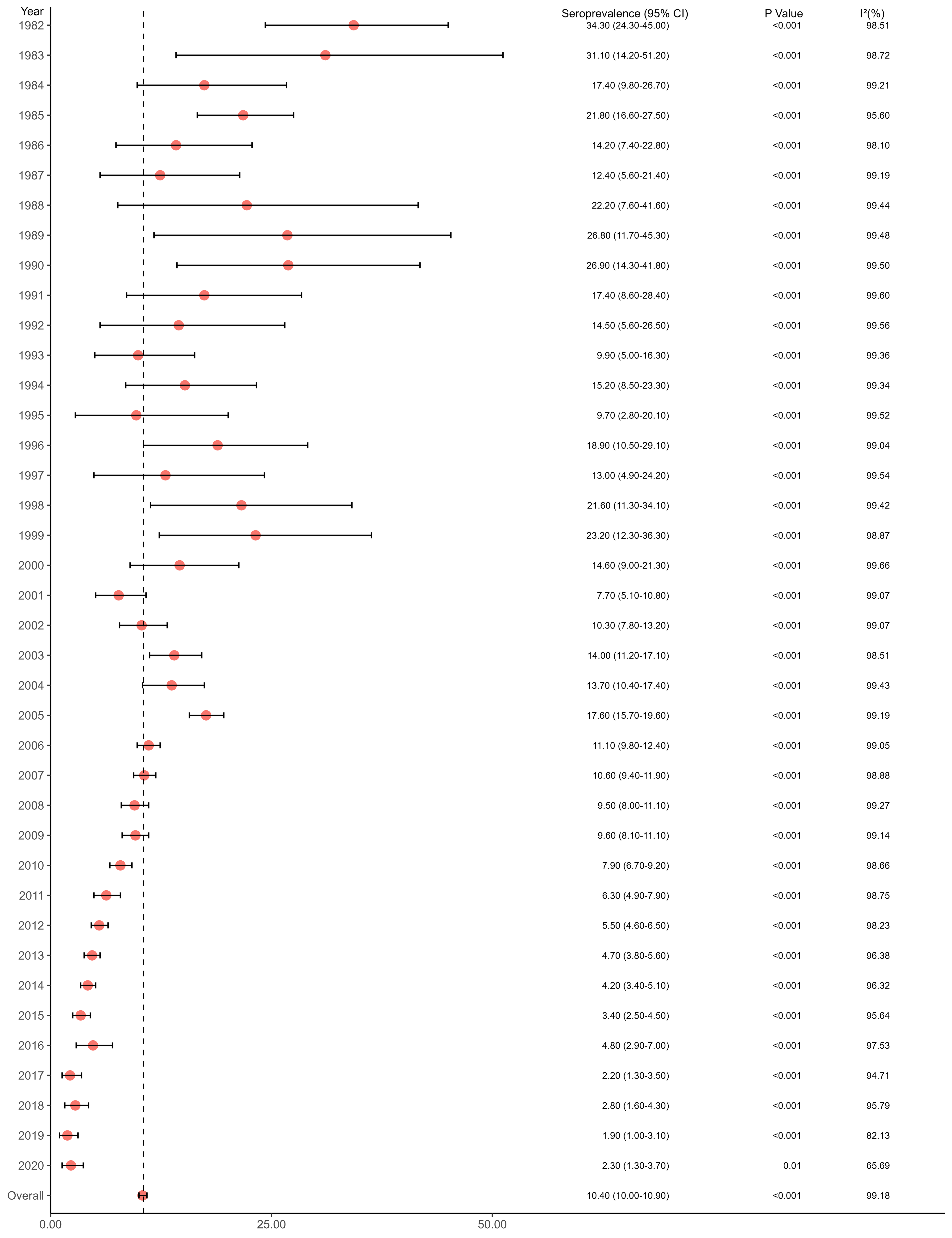

Supplement: S2 Fig — (TIF) [file pntd.0012466.s004.tif]

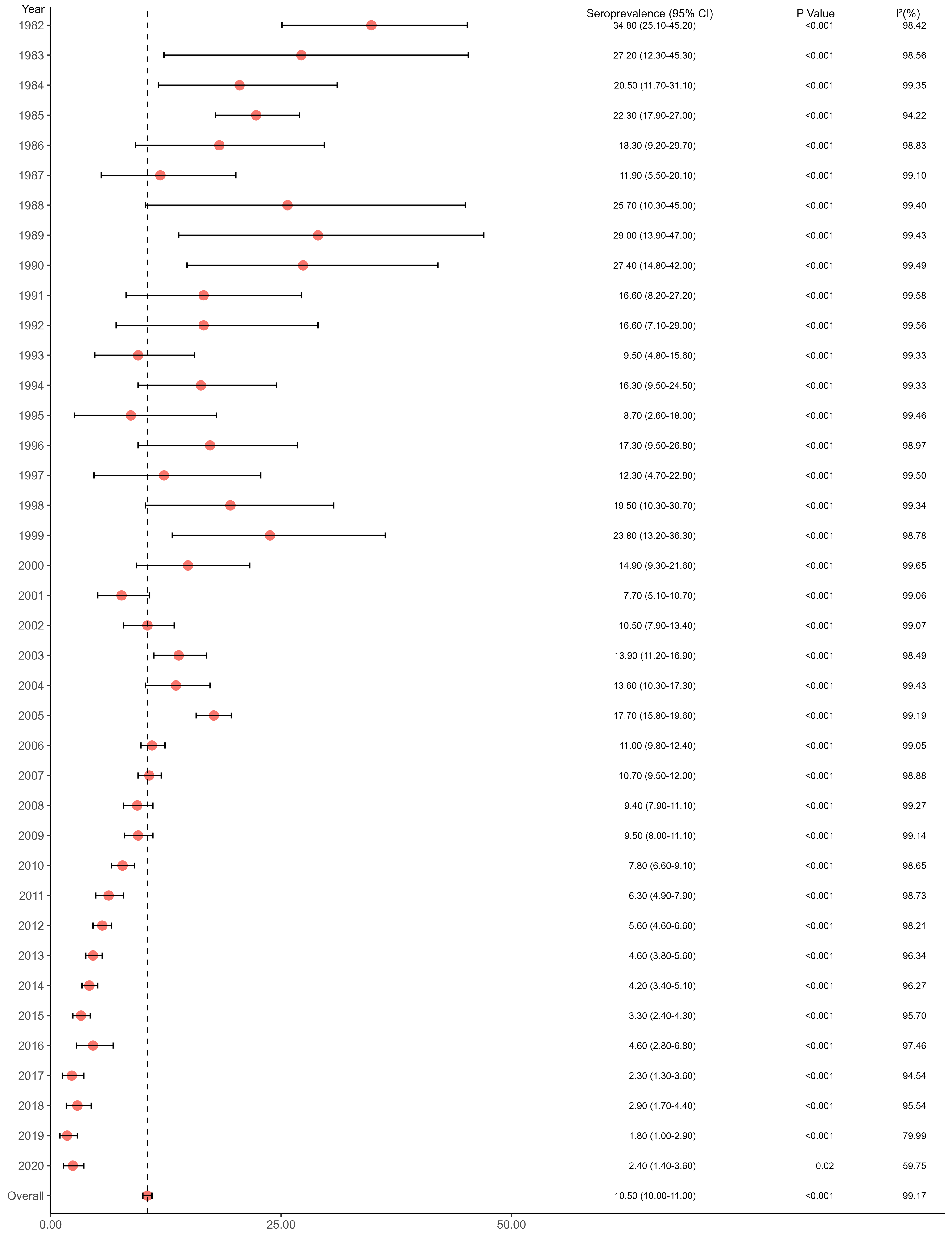

Supplement: S3 Fig — (TIF) [file pntd.0012466.s005.tif]
